# Overexpression of ceramide synthase 1 increases C18-ceramide and leads to lethal autophagy in human glioma

**DOI:** 10.18632/oncotarget.21955

**Published:** 2017-10-23

**Authors:** Zheng Wang, Lijun Wen, Fei Zhu, Yanping Wang, Qing Xie, Zijun Chen, Yunsen Li

**Affiliations:** ^1^ Institutes of Biology and Medical Sciences, Medical College, Jiangsu Institute of Hematology, The First Affiliated Hospital of Soochow University, Soochow University, Suzhou, China; ^2^ Shanghai University of Traditional Chinese Medicine, Shanghai, China

**Keywords:** ceramide synthase 1, C18-ceramide, glioma, mass spectrometry, autophagy

## Abstract

Ceramide synthase 1 (CERS1) is the most highly expressed CERS in the central nervous system, and ceramide with an 18-carbon–containing fatty acid chain (C18-ceramide) in the brain plays important roles in signaling and sphingolipid development. However, the roles of CERS1 and C18-ceramide in glioma are largely unknown. In the present study, measured by electrospray ionization linear ion trap mass spectrometry, C18-ceramide was significantly lower in glioma tumor tissues compared with controls (*P* < 0.001), indicating that C18-ceramide might have a role in glioma. These roles were examined by reconstitution of C18-ceramide in U251 and A172 glioma cells via addition of exogenous C18-ceramide or overexpression of CERS1, which has been shown to specifically induce the generation of C18-ceramide. Overexpression of CERS1 or adding exogenous C18-ceramide inhibited cell viability and induced cell death by activating endoplasmic reticulum stress, which induced lethal autophagy and inhibited PI3K/AKT signal pathway in U251 and A172 glioma cells. Moreover, overexpression of CERS1 or adding exogenous C18-ceramide increased the sensitivity of U251 and A172 glioma cells to teniposide (VM-26). Thus, the combined therapy of CERS1/C18-ceramide and VM-26 may be a novel therapeutic strategy for the treatment of human glioma.

## INTRODUCTION

Gliomas are a group of heterogeneous primary tumors in the central nervous system (CNS). Malignant gliomas are the most common primary brain tumors in the CNS and account for approximately 79% of malignant CNS tumors [1]. In general, patients with anaplastic astrocytoma have a 3-year survival time, and glioblastoma multiforme is even worse, with less than 12 months [2]. Although the development of technology have made great progress in the diagnosis and therapy of glioma, problems still remain because of drug resistance during chemotherapy [3–5].

Sphingolipids perform essential functions in all eukaryotes and are major players in cell signaling, growth, apoptosis, differentiation, and proliferation [6, 7]. Ceramide, a hydrophobic backbone of sphingolipids, contains two hydrophobic chains, a long-chain base and a fatty acid, which are connected via an amide bond [8]. Ceramide synthase (CERS), important enzymes in the ceramide synthesis process, acetylate sphinganine to dihydroceramide with different chain lengths, varying in lengths from C14 to C26 [9, 10]. There are six CERS isoforms in mammalian cells (CERS1-6), which exert specificity for the generation of ceramides with distinct fatty acid chain lengths. CERS1 generates mainly ceramide with an 18-carbon–containing fatty acid chain (C18-ceramide) [11, 12].

In the CNS, the most highly expressed CERS is CERS1, which is particularly present in neurons of the neocortex, hippocampus, and cerebellum [13]. Reduction of C18-ceramide and its derivatives in the brain leads to neurodegeneration in mice and myoclonus epilepsy with dementia in humans [7]. In addition, C18-ceramide causes apoptosis in certain cancer cells [14]. For example, endogenous C18-ceramide levels are significantly decreased in the majority of human head and neck squamous cell carcinoma (HNSCC) tissues as compared with those in normal tissues [15]. Furthermore, CERS1 and C18-ceramide have emerged as tumor suppressors in preclinical and clinical studies [16–18].

Endoplasmic reticulum (ER) stress is induced by various intracellular and extracellular factors that disturb any homeostatic functions, including protein synthesis, lipid formation, Ca^2+^ storage, and signaling in the ER [19–22]. Upon ER stress, GRP78/BIP binds to the misfolded proteins and then dissociates itself from these sensors: PKR-like ER kinase (PERK), inositol-requiring kinase (1IRE1), and activating transcription factor 6 (ATF-6); this dissociation subsequently activates the sensors [23]. As a consequence, PERK becomes activated by autophosphorylation and subsequently triggers inhibition of protein translation by phosphorylation of eukaryotic translation initiation factor 2α (eIF2α), which decreases mRNA translation to stop the accumulation of protein in the ER [24]. In addition, such activation allows for ATF-4, X-box binding protein 1 (XBP-1), and ATF-6 to translocate to the nucleus and increase transcription of C/EBP homologous protein (CHOP) and other genes that are associated with ER stress–mediated apoptosis [25, 26]. LC3B-II is a good marker of autophagy because it can bind to the inner autophagosome membrane [27]. ER stress negatively regulates the AKT/TSC/mTOR pathway to enhance autophagy [28]. Insulin-like growth factor 1 (IGF-1) is one of the most potent natural activators of the PI3K/AKT signaling pathway, and it is a stimulator of cell growth and proliferation.

In the present study, we used electrospray ionization linear ion trap mass spectrometry (ESI-LIT-MS) to detect the ceramide expression profiles from human brain tissues of glioma patients and controls. C18-ceramide was significantly lower in glioma tumor tissues compared with controls (*P* < 0.001). Further evidence was present to establish the role of CERS1/C18-ceramide in the inhibition of cell viability and the induction of cell death; these mechanisms might be the modulation of ER stress, induction of lethal autophagy, and inhibition of the PI3K/AKT signaling pathway in glioma cells *in vitro*. Here, we further reported that CERS1/C18-ceramide in combination with teniposide (VM-26) displayed an even greater inhibitory effect on the U251 and A172 glioma cells than did VM-26 alone.

## RESULTS

### C18-ceramide expression pattern in glioma patients and controls

**Figure 1:**
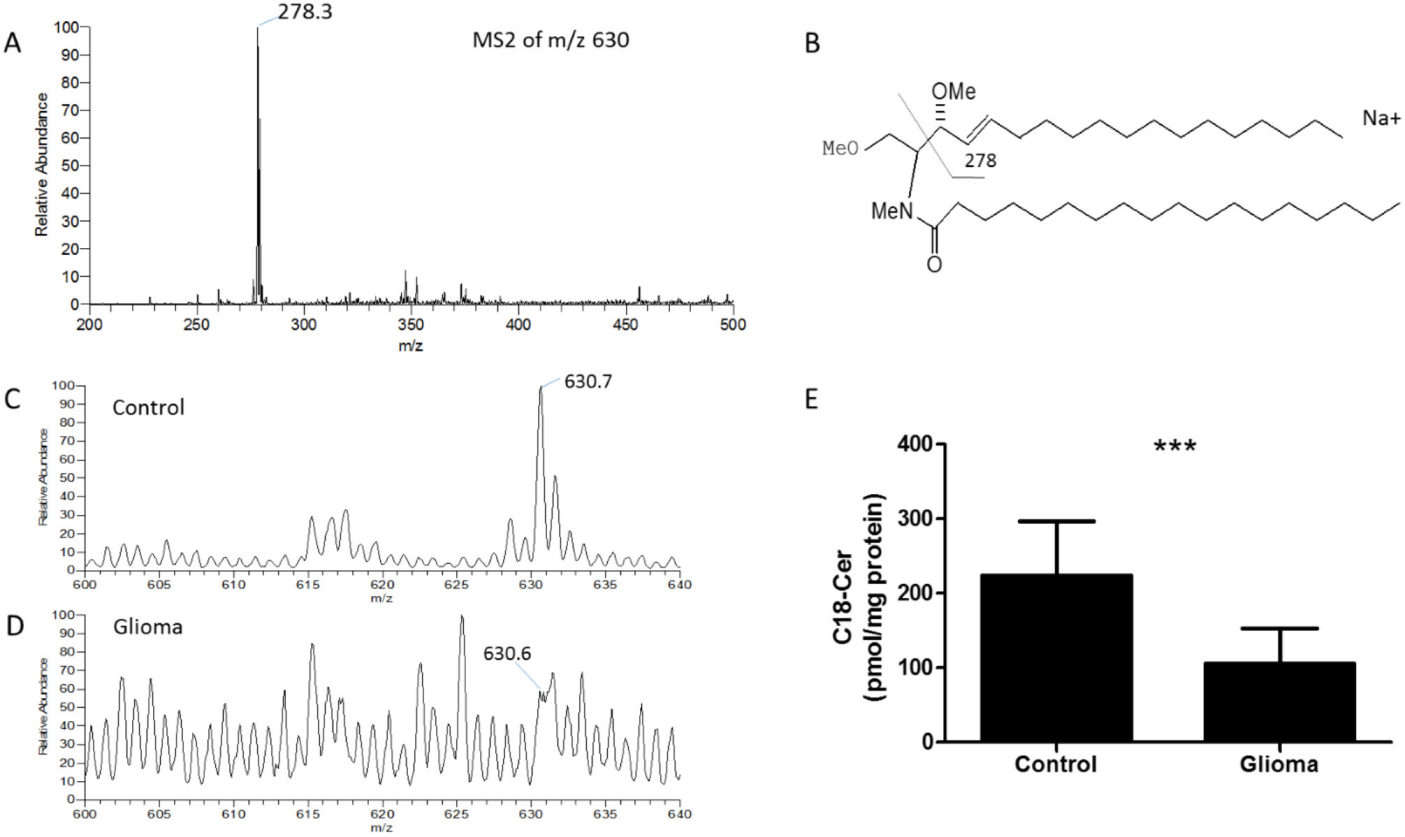
Qualitative and quantitative analysis of C18-ceramide in human glioma tissue samples **(A)** MS2 spectrum of m/z 630, characteristic fragmentation products (m/z 278.3) for permethylated C18-ceramide in the human glioma tissue samples in positive-mode MS2. **(B)** Fragmentation pathway and characteristic decomposition products for permethylated C18-ceramide in the positive mode. **(C)** MS1 profile of C18-ceramide isolated from a control tissue sample; the expression of C18-ceramide (m/z 630) was high. **(D)** MS1 profile of C18-ceramide isolated from a glioma tissue sample; the expression of C18-ceramide (m/z 630) was low. **(E)** Relative quantification of C18-ceramide (m/z 630) in the tissue samples of controls and glioma. Data represent the tissue samples from controls (n = 5) and glioma (n = 14). Statistical significance between glioma and controls was analyzed using the two-tailed Student’s t-test of means. Compared with control, *** *P* < 0.001.

We used ESI-LIT-MS to analyze the structure of ceramides extracted from human brain tissue samples. A number of potential molecular ions (as Na^+^ adducts) were found in the ESI-LIT-MS^1^ profile spectrum isolated from the samples. To identify the exact structures of those ions, we systematically acquired MS^2^ spectra of every molecular precursor ion in the MS^1^ spectrum. The presence of methylated C18-ceramide was affirmed by the analysis of the MS^2^ pathway in the positive mode, in which the parent ion was m/z 630 (Figure 1A). m/z 278 was the key fragment of the d18:1/18:0 ceramide (m/z 630) (Figure 1B). We used a method of relative quantification to analyze the C18-ceramide. C18-ceramide expression was higher in control than in glioma (*P* < 0.001) (Figure 1C-1E). The amount of this ceramide in the tumor site might be important for its regulatory roles in the glioma. Furthermore, the C16-ceramide was increased in glioma, and the sphingosine was decreased in glioma. But C14-creamide, C20-ceramide, C24-ceramide and sphingosine 1 phosphate (S1P) in glioma had no significant difference compared with control (Supplementary Figure 1A-1F).

### Overexpression of CERS1 or exogenous of C18-ceramide reduces cell viability and induces cell death in U251 and A172 glioma cells

**Figure 2:**
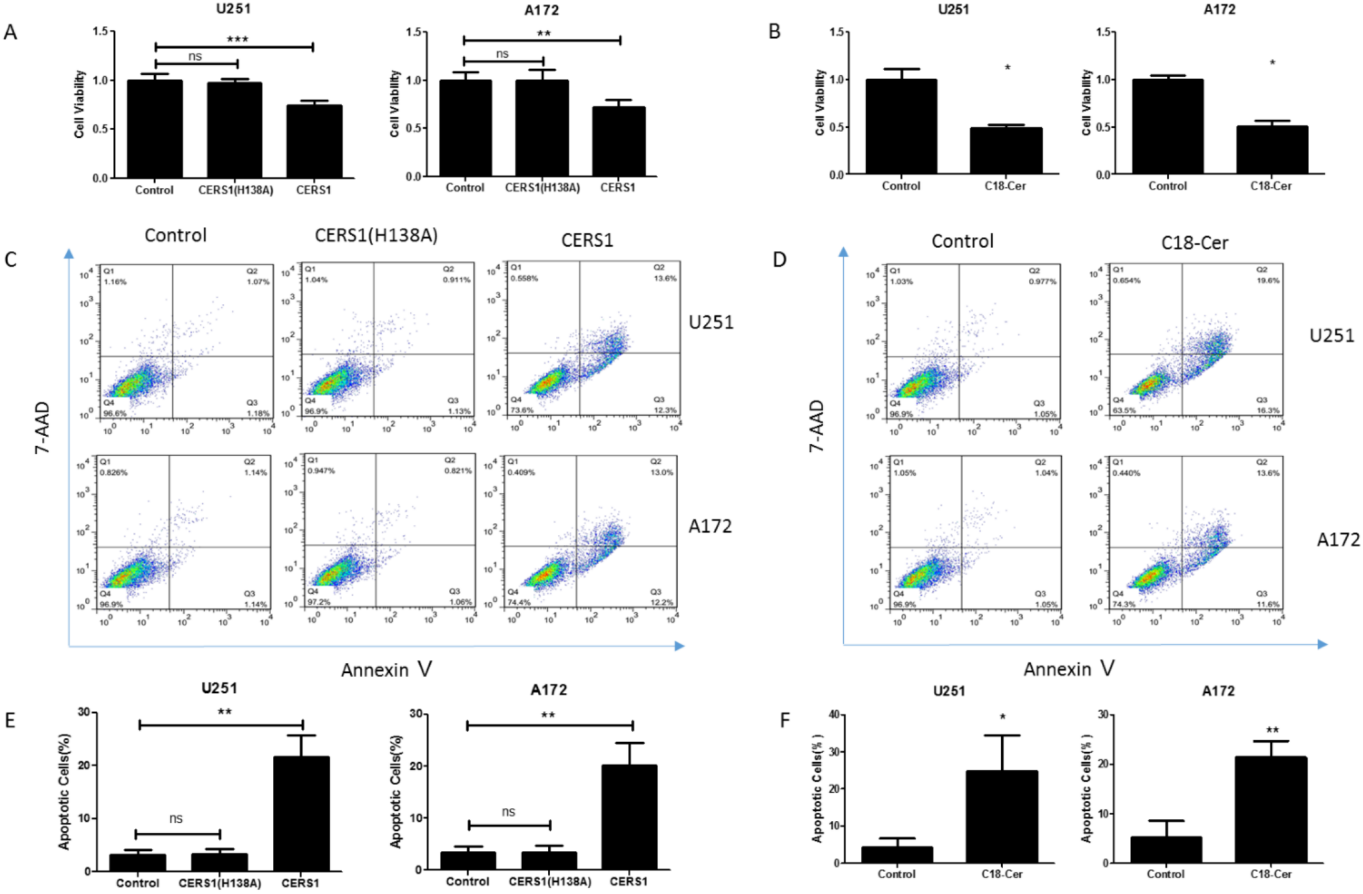
Inhibition of cell viability and promotion of cell death induced by overexpression of CERS1 and exogenous C18-ceramide in U251 and A172 glioma cells **(A)** Effect of catalytically inactive CERS1 (H138A) and CERS1 overexpression on the cell viability of U251 and A172 cells for 48h. **(B)** Effect of exogenous C18-ceramide (20 μM) on the cell viability of U251 and A172 cells for 48h. **(C)** Effect of catalytically inactive CERS1 (H138A) and CERS1 overexpression on the cell death of U251 and A172 cells for 48h. **(D)** Effect of exogenous C18-ceramide (20 μM) on the cell death of U251 and A172 cells for 48h. **(E)** Quantitative analysis of catalytically inactive CERS1 (H138A) and CERS1 overexpression on the cell death of U251 and A172 cells for 48h. **(F)** Quantitative analysis of exogenous C18-ceramide (20 μM) on the cell death of U251 and A172 cells for 48h. Statistical significance between CERS1/C18-ceramide and controls was analyzed using the two-tailed Student’s t-test of means. Values represent the means ± SD, n = 3 independent experiments. Compared with control, *P < 0.05, **P < 0.01.

To determine the functions of C18-ceramide in glioma, we increased the expression of CERS1 and CERS1 (H138A) by pcDNA3.1(+)/CERS1 transfection, which exclusively synthesized C18-ceramide, in glioma cells U251 (Supplementary Figure 2A, 2C) and A172 (Supplementary Figure 2B, 2D). The effects of overexpression of CERS1 on cell viability were examined using a CCK-8 assay. CERS1 expression reduced cell viability compared with controls (Figure 2A). But, knock down of CERS1 (CERS1 RNAi) had no effect on the cell viability of U251 and A172 cells (Supplementary Figure 5A). We then examined the roles of exogenous C18-ceramide by C18-ceramide treatment (20 μM, for 48h) in the regulation of cell viability. Using the CCK-8 assay, we observed similar results of C18-ceramide also decreasing cell viability (Figure 2B). Furthermore, the R (+/-) Methanandamide also decreased the cell viability (Supplementary Figure 5B).

Induction of cell death was further confirmed by flow cytometry analysis after Annexin V/FITC and 7-AAD staining. Results from the assays clearly showed that overexpression of CERS1 strongly induced cell death (Figure 2C, 2E). C18-ceramide also could induce cell death, as detected by Annexin V/7-AAD assay (Figure 2D, 2F). Furthermore, the same results were existed in U87 and U118 glioma cells (Supplementary Figure 3A-3D). These data suggested a new role for the tumor suppressors CERS1 and C18-ceramide in decreasing cell viability and increasing cell death.

### Activation of ER stress induced by overexpression of CERS1

**Figure 3:**
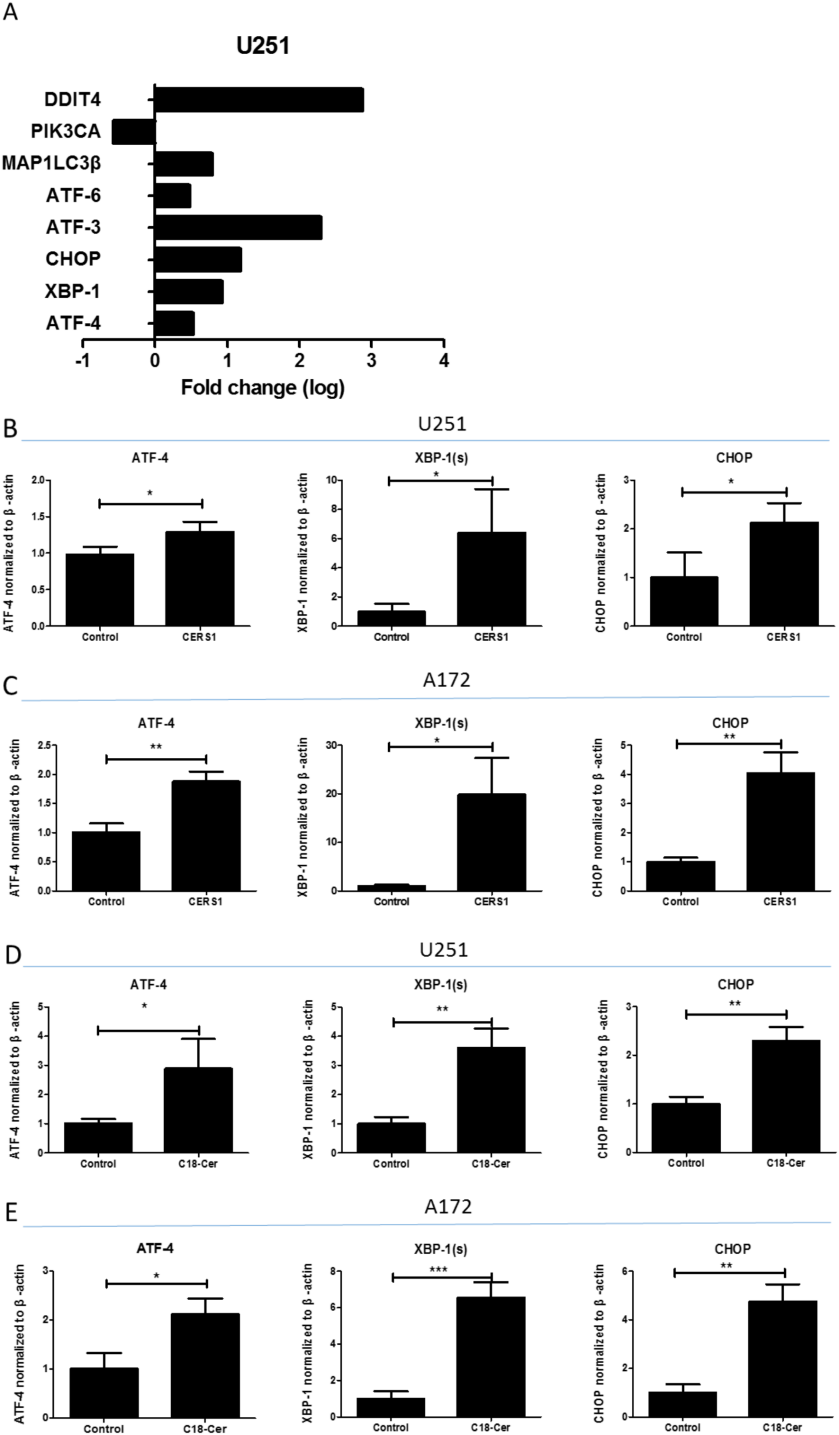
Activation of ER stress induced by overexpression of CERS1 in U251 and A172 glioma cells **(A)** The fold change of DDIT4, PIK3CA, MAP1LC3β, ATF-6, ATF-3, CHOP, XBP-1, and ATF-4 in CERS1 overexpression in U251 cells compared with controls. **(B)** The qRT-PCR results of ATF-4, XBP-1 (s), and CHOP mRNA levels in CERS1 overexpression in U251 cells compared with controls. **(C)** The qRT-PCR results of ATF-4, XBP-1 (s), and CHOP mRNA levels in CERS1 overexpression in A172 cells compared with controls. **(D)** The qRT-PCR results of ATF-4, XBP-1 (s), and CHOP mRNA levels in exogenous C18-ceramide (20 μM) in U251 cells compared with controls. **(E)** The qRT-PCR results of ATF-4, XBP-1 (s), and CHOP mRNA levels in exogenous C18-ceramide (20 μM) in A172 cells compared with controls. Statistical significance between CERS1/C18-ceramide and controls was analyzed using the two-tailed Student’s t-test of means. Values represent the means ± SD, n = 3 independent experiments. Compared with control, *P < 0.05, **P < 0.01, ***P < 0.001.

We next examined the potential signaling pathways that might induce the cell viability reduction and cell death via overexpression of CERS1 or addition of exogenous of C18-ceramide in glioma cells. In the gene expression microarray, DDIT4 (a negative regulator of mTOR), PIK3CA, MAP1LC3β, ATF-6, ATF-3, CHOP, XBP-1, and ATF-4 had changes in U251 cells overexpressing CERS1 compared with controls (Figure 3A). These data demonstrated that CERS1/C18-ceramide might modulate the ER stress, autophagy, and PI3K/AKT pathways. We also verified these results using qRT-PCR analysis; the mRNA levels of ATF-4, XBP-1 (s), and CHOP obviously increased in U251 and A172 glioma cells in which CERS1 was overexpressed (Figure 3B, 3C). And, the mRNA levels of ATF-4, XBP-1 (s), and CHOP were increased upon C18-ceramide addition in U251 and A172 glioma cells (Figure 3D, 3E).

**Figure 4:**
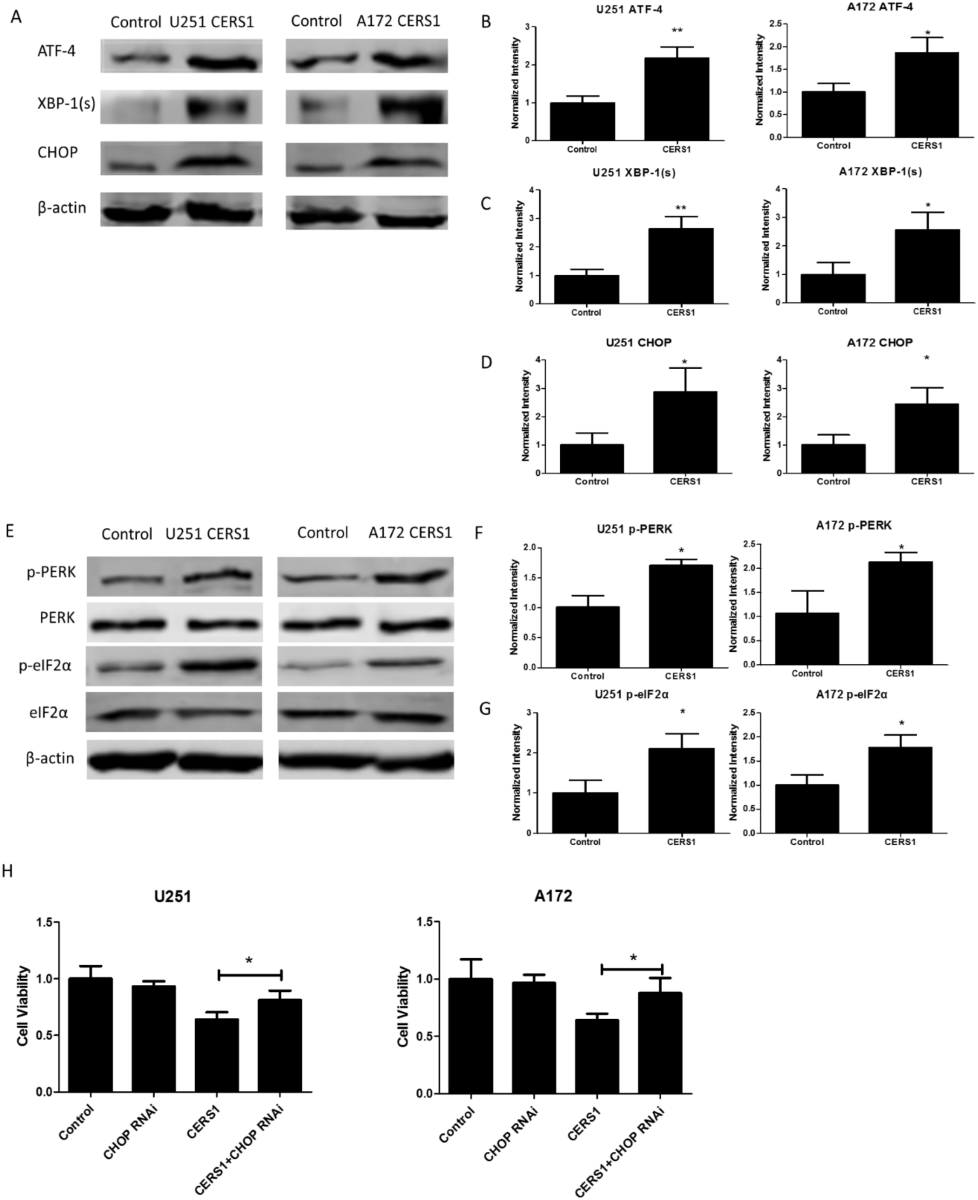
Activation of ER stress induced by overexpression of CERS1 **(A)** Western blot analysis was performed to detect the expression levels of ATF-4, XBP-1 (s), and CHOP and β-actin in U251 and A172 cells after overexpression of CERS1 compared with controls. **(B)** ATF-4 signals were normalized to β-actin for relative quantification in U251 and A172 cells after overexpression of CERS1 compared with controls. **(C)** XBP-1 (s) signals were normalized to β-actin for relative quantification in U251 and A172 cells after overexpression of CERS1 compared with controls. **(D)** CHOP signals were normalized to β-actin for relative quantification in U251 and A172 cells after overexpression of CERS1 compared with controls. **(E)** Western blot analysis was performed to detect the expression levels of p-PERK, PERK, p-eIF2α, eIF2α, and β-actin in U251 and A172 cells after overexpression of CERS1 compared with controls. **(F)** p-PERK signals were normalized to PERK for relative quantification in U251 and A172 cells after overexpression of CERS1 compared with controls. **(G)** p-eIF2α signals were normalized to eIF2α for relative quantification in U251 and A172 cells after overexpression of CERS1 compared with controls. **(H)** Effect of loss of CHOP function (using CHOP siRNA) on cell viability of overexpression of CERS1 in U251 and A172 cells for 48h. Statistical significance was analyzed using the two-tailed Student’s t-test of means. Values represent the means ± SD, n = 3 independent experiments. *P < 0.05, **P < 0.01.

Furthermore, the protein expression levels of ATF-4, XBP-1 (s), and CHOP were also validated. By Western blot, the protein levels of ATF-4, XBP-1 (s), and CHOP increased in U251 and A172 glioma cells in which CERS1 was overexpressed (Figure 4A, 4B, 4C, 4D). The elevation of p-PERK and p-eIF2α also indicated CERS1-induced ER stress in U251 and A172 glioma cells (Figure 4E, 4F, 4G). Loss of CHOP function (using CHOP siRNA) (Supplementary Figure 4A) recovered the depressed cell viability caused by overexpression of CERS1 (Figure 4H). Furthermore, CHOP knockdown could weaken the autophagy induction (Supplementary Figure 4A). And tunicamycin (potentiate ER stress) could strengthen the decrease on cell viability combined with CERS1 in U251 and A172 cells (Supplementary Figure 5C). In conclusion, overexpression of CERS1 in glioma cells induced activation of ER stress.

### CERS1 induces lethal autophagy in U251 and A172 cells

**Figure 5:**
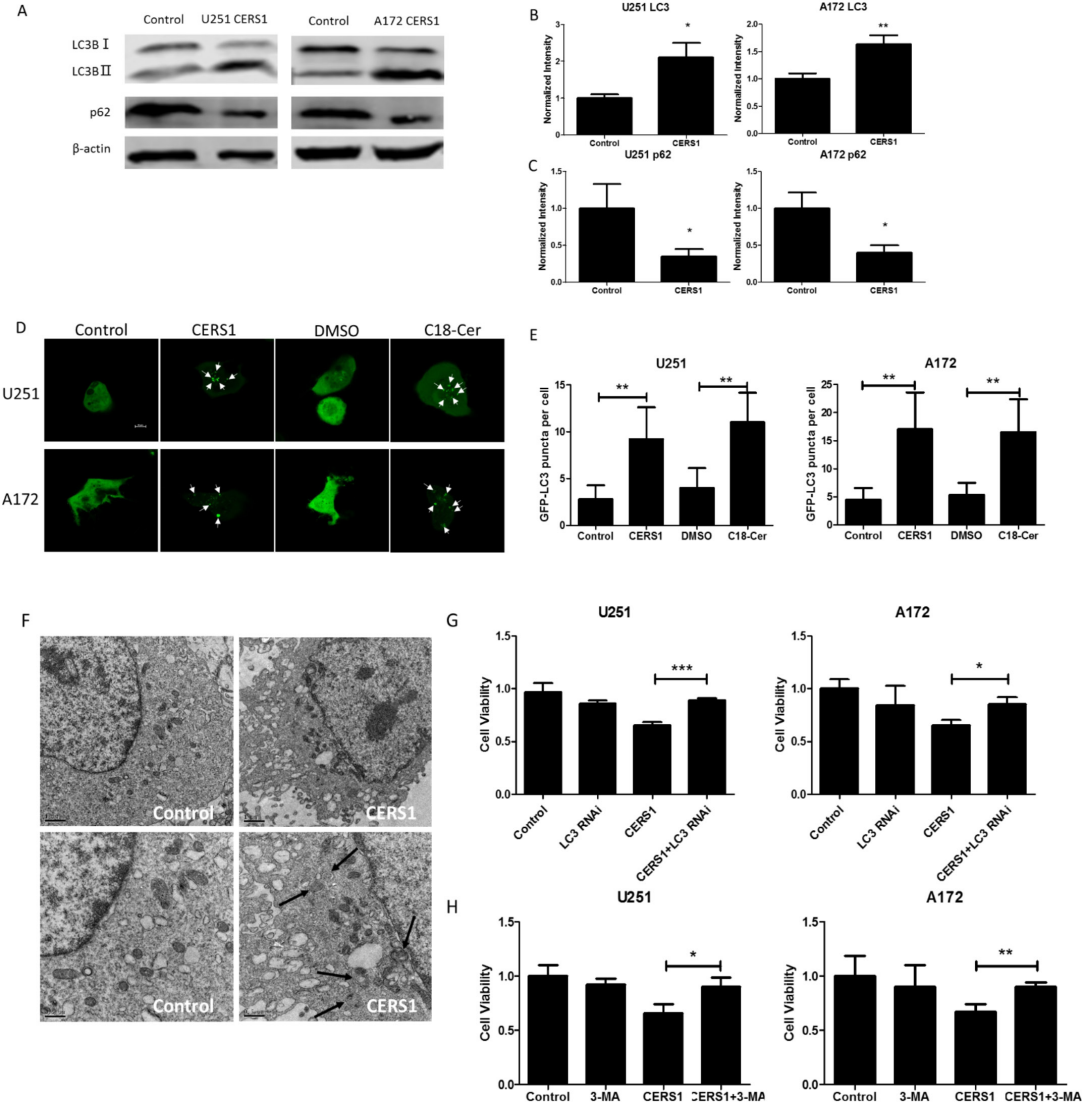
Lethal autophagy induced by overexpression of CERS1 in U251 and A172 cells **(A)** Western blot analysis was performed to detect the expression levels of LC3B, p62, and β-actin in U251 and A172 cells after overexpression of CERS1. **(B)** LC3B-II signals were normalized to LC3B-I for relative quantification in U251 and A172 cells after overexpression of CERS1 compared with controls. **(C)** p62 signals were normalized to β-actin for relative quantification in U251 and A172 cells after overexpression of CERS1 compared with controls. **(D)** Overexpression of CERS1 or exogenous C18-ceramide (20 μM) increased the number of GFP–LC3 puncta (arrows) were visualized using confocal microscopy in U251 and A172 cells. Scale bar = 10 μm. **(E)** Quantification of GFP puncta per cell were performed in U251 and A172 cells. **(F)** Formation of double-membrane autophagosomal vesicles (black arrows) in the control or overexpression of CERS1 was visualized by TEM (left and right, respectively) in U251 cells. Higher magnification of TEM visualization is shown in lower panels. Scale bars, 1 μm (top) and 0.5 μm (bottom). **(G)** Effect of loss of LC3 function (using LC3 siRNA) on cell viability of overexpression of CERS1 in U251 and A172 cells for 48h. **(H)** 3-MA (5 mM) effect on cell viability of overexpression of CERS1 in U251 and A172 cells for 48h. Statistical significance was analyzed using the two-tailed Student’s t-test of means. Values represent the means ± SD, n = 3 independent experiments. *P < 0.05, **P < 0.01.

We examined the expression of LC3 and p62, which are indicators of autophagy. As a result, the conversion of LC3B-I (upper band) to LC3B-II (lower band) and reduction of p62 reflected the occurrence of autophagy in U251 and A172 glioma cells in which CERS1 was overexpressed (Supplementary Figure 5A-5C). Overexpression of CERS1 and exogenous of C18-ceramide also significantly increased the punctate distribution and density of GFP-LC3 in U251 and A172 cells (Figure 5D, 5E). CERS1-mediated autophagy in U251 cells was also visualized by TEM, which showed that overexpression of CERS1 increased the formation of autophagosomes compared to control (Figure 5F). We further investigated whether the inhibitory effect of CERS1 on cell viability was associated with the induced autophagy. Cells were silenced of LC3 or pretreated with autophagy inhibitor 3-MA (Supplementary Figure 4C). As shown in Figure 5G and 5H, the cell viability was significantly increased in the CERS1+LC3 RNAi group and CERS1+3-MA group compared with the CERS1 group in U251 and A172 cells, suggesting that LC3 RNAi or 3-MA blocked the inhibitory effect of CERS1 on cell viability. Further, the autophagic flux inhibitor Bafilomycin A1 (BA1) could lead to LC3 accumulation in CERS1 overexpression group in U251 and A172 cells (Supplementary Figure 5D). The results also indicated that CERS1 induced lethal autophagy in U251 and A172 cells.

### CERS1 inhibits PI3K/AKT pathway in U251 and A172 cells

**Figure 6:**
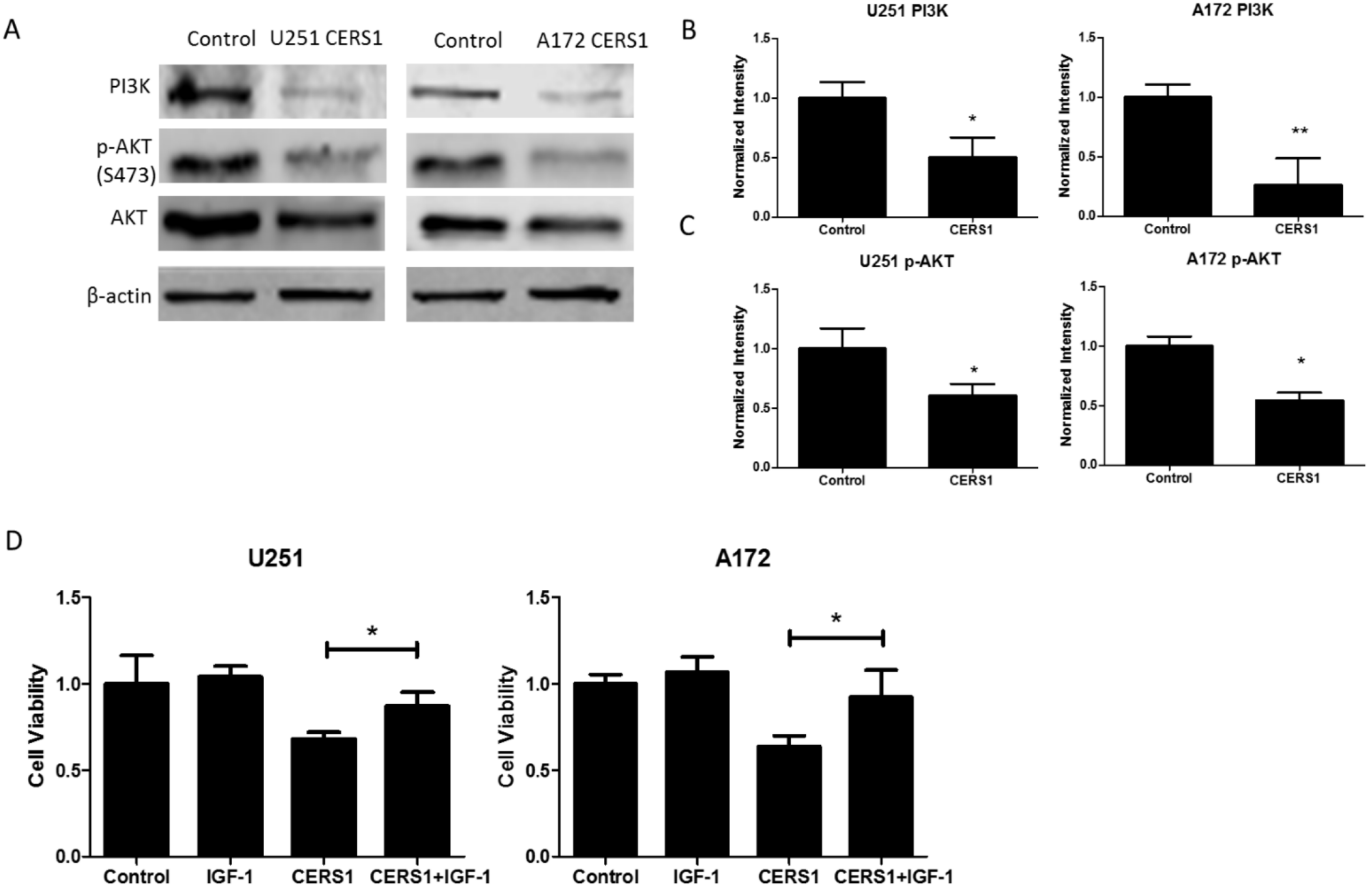
CERS1 inhibits PI3K/AKT pathway in U251 and A172 cells **(A)** Western blot analysis was performed to detect the expression levels of PI3K, p-AKT (S473), AKT, and β-actin in U251 and A172 cells after overexpression of CERS1. **(B)** PI3K signals were normalized to β-actin for relative quantification in U251 and A172 cells after overexpression of CERS1 compared with controls. **(C)** p-AKT (S473) signals were normalized to AKT for relative quantification in U251 and A172 cells after overexpression of CERS1 compared with controls. **(D)** Effect of IGF-1 (20 ng/mL) on cell viability of overexpression of CERS1 in U251 and A172 cells for 48h. Statistical significance was analyzed using the two-tailed Student’s t-test of means. Values represent the means ± SD, n = 3 independent experiments. *P < 0.05, **P < 0.01.

Considering the already known relation between the PI3K/AKT pathway and autophagy, we explored whether CERS1 induces ER stress and autophagy through the suppression of the PI3K/AKT pathway. The expression of PI3K and p-AKT (S473) were decreased in the CERS1 group compared with the control group (Figure 6A-6C). In addition, cell viability in the CERS1+IGF-1 (Supplementary Figure 4B) group was increased compared with the CERS1 group in U251 and A172 cells (Figure 6D). These results suggest that IGF-1 blocked the effect of CERS1 on the reduction of cell viability. Taken together, the suppression of PI3K/AKT pathway activity contributed to CERS1-induced cell viability reduction.

### Overexpression of CERS1 or exogenous of C18-ceramide increases the chemosensitivity to VM-26 in U251 and A172 glioma cells

**Figure 7:**
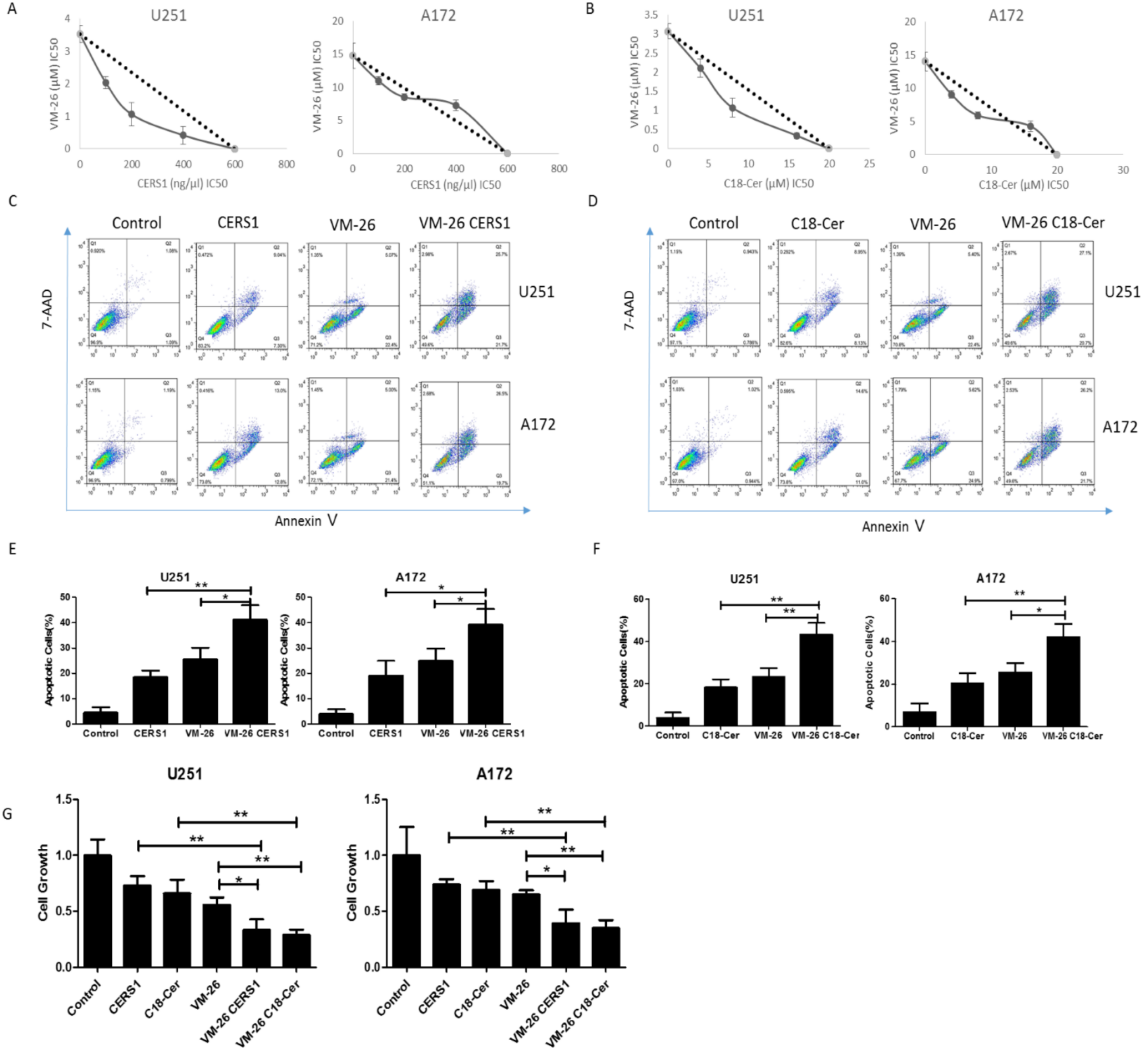
Overexpression of CERS1 and exogenous C18-ceramide increases the chemosensitivity to VM-26 in U251 and A172 glioma cells **(A)** Synergistic interactions of overexpression of CERS1 and VM-26 in the inhibition of cell viability were examined by quantitative isobologram studies. The IC50 concentrations of CERS1 plasmid in the presence of increasing concentrations of VM-26 were determined by CCK-8 assays, and the data were plotted in isobolograms. A straight line joining points on x- and y-axes represents the IC50 concentrations of transfection CERS1 plasmid and VM-26 alone. The points on the isobologram representing the IC50 values of VM-26 obtained in the transfection of 100, 200, and 400 ng/μl CERS1 plasmid fell within the left of the straight line, which indicates synergism. **(B)** Synergistic interactions of exogenous C18-ceramide and VM-26 in the inhibition of cell viability were examined by quantitative isobologram studies. The IC50 concentrations of exogenous C18-ceramide in the presence of increasing concentrations of VM-26 were determined by CCK-8 assays, and the data were plotted in isobolograms. A straight line joining points on x- and y-axes represents the IC50 concentrations of exogenous C18-ceramide and VM-26 alone. The points on the isobologram representing the IC50 values of VM-26 obtained in the present of 4, 8, and 16 μM exogenous C18-ceramide fell within the left of the straight line, which indicates synergism. **(C)** Effect of CERS1 overexpression and VM-26 treatment on the cell death of U251 (0.25 μM VM-26) and A172 (10 μM VM-26) cells for 48h. **(D)** Effect of exogenous C18-ceramide (20 μM) and VM-26 treatment on the cell death of U251 (0.25 μM VM-26) and A172 (10 μM VM-26) cells for 48h. **(E)** Quantitative analysis of CERS1 overexpression and VM-26 treatment on cell death of U251 (0.25 μM VM-26) and A172 (10 μM VM-26) cells for 48h. **(F)** Quantitative analysis of exogenous C18-ceramide (20 μM) on cell death of U251 (0.25 μM VM-26) and A172 (10 μM VM-26) cells for 48h. **(G)** Cell growth of control, VM-26 group, VM-26/CERS1 group and VM-26/C18-ceramide (20 μM) group in U251 (0.25 μM VM-26) and A172 (10 μM VM-26) cells for 48h detected by BrdU incorporation assay. Statistical significance between VM-26 CERS1/C18-ceramide group and VM-26 group was analyzed using the two-tailed Student’s t-test of means. Values represent the means ± SD, n = 3 independent experiments. Compared with VM-26, *P < 0.05, **P < 0.01, ***P < 0.001.

VM-26, a semisynthetic derivative of podophyllotoxin resin, is a cell cycle–specific cytotoxic drug, damaging DNA and inducing cellular apoptosis [29]. Overexpression of CERS1 or adding exogenous of C18-ceramide reduced glioma cell viability, at least partially by enhancing cell death. This enhanced cell death by overexpression of CERS1 or exogenous of C18-ceramide prompted us to investigate whether U251 and A172 cells receiving such treatments were also more sensitive to the antitumor drug VM-26. The effects of overexpression of CERS1 and treatment of VM-26 on cell viability inhibition were examined using a CCK-8 assay. As seen in Figure 7A, treatment with CERS1 in the presence of increasing concentrations of VM-26 for 48h synergistically inhibited the growth of U251 cells. Interestingly, the combination of VM-26 with higher concentrations (400 ng/μl) of CERS1 showed antagonism. Then, by Annexin V/7-AAD assay, the group treated with both CERS1 overexpression and VM-26 also increased the amount of apoptosis cells compared with the group treated with VM-26 alone (Figure 7C, 7E). Similar results showed that adding exogenous C18-ceramide had the same effect in combination with VM-26 by CCK-8 and Annexin V/7-AAD assays (Figure 7B, 7D, 7F). In addition, a BrdU incorporation assay was used to determine cell growth. VM-26 remarkably reduced the proliferation of both U251 and A172 cells. Furthermore, overexpression of CERS1 or adding exogenous C18-ceramide combined with VM-26 induced a stronger reduction of cell growth in U251 and A172 cells (Figure 7G). Thus, CERS1 and C18-ceramide potentially delayed the growth of gliomas cells and inhibited the DNA synthesis. But, these combination treatment did not affect the C18-ceramide level, as well as the induction of ER stress and autophagy compared with CERS1 alone (Supplementary Figure 6A-6D). Taken together, overexpression of CERS1 or adding exogenous of C18-ceramide increased the sensitivity of U251 and A172 glioma cells to VM-26.

## DISCUSSION

In our study, decreased levels of C18-ceramide might play important roles in glioma. By MS, C18-ceramide was lower in glioma tumor tissues compared with controls. Further data showed that increased generation of C18-ceramide via overexpression of CERS1 or adding exogenous of C18-ceramide resulted in the inhibition of cell viability, which involved the activation of ER stress, induction of autophagy, and modulation of the PI3K/AKT pathway in glioma cells. These results suggested that decreased levels of C18-ceramide imparted a growth advantage to cancer cells, whereas increased generation of C18-ceramide could lead to inhibition of glioma.

The application of ESI and matrix-assisted laser desorption ionization, in conjunction with tandem collision-induced dissociation, time of flight, and quadrupole ion-trap techniques, has revolutionized the sensitivity of MS [30–33]. Our study has greatly promoted the applicability of the MS technology for analysis of patient samples. Our method was based on the characteristic fragmentations of the ceramides [34]. Gliomas make up approximately 30% of all brain and CNS tumors and 80% of all malignant brain tumors [35]. In this study, C18-ceramide was lower in glioma tumor tissues compared with controls, whereas there was no significant difference in some benign brain tumors (such as meningioma and hypophysoma) (Supplementary Figure 7).

Ceramides are the precursors for complex sphingolipids and can be metabolized to complex sphingolipids [36]. Ceramide metabolism involves complex processes, and each CERS isoform exhibits a preference for a fatty acyl CoA of defined chain length acting as substrate [37, 38]. For example, CERS1 and CERS4 generate mainly C18-ceramide, whereas CERS5 and CERS6 generate mainly C16-ceramide [39, 40]. Meanwhile, the ceramides with different chain length generated by distinct CERS have opposite roles in cell proliferation and death. In HNSCC cells, CERS1 overexpression or C18-ceramide treatment suppresses tumor growth, but CERS6 downregulation produces ER stress leading to apoptosis of cells [41–43]. In our study, overexpression of CERS1 also inhibited glioma cell viability and induced cell death. Interestingly, combined with the overexpression of CERS1 in glioma cells, the mRNA level of CERS4 was also increased (Supplementary Figure 8A, 8B). Conversely, the mRNA level of CERS6 was significantly decreased in CERS1 overexpressed glioma cells (Supplementary Figure 8C, 8D). These results indicate that the C18-ceramide generated by CERS1 has diverse roles compared with C16-ceramide generated by CERS6 in glioma cells.

ER stress can occur earlier than the induction of autophagy and trigger PI3K/Akt/mTOR signaling pathway inactivation both *in vivo* and *in vitro* [44]. When ER stress is moderate and recoverable, cells may activate the PI3K/Akt/mTOR signaling pathway through unfolded protein response (UPR) to transmit survival signals and overcome the adverse condition. In comparison, when ER stress is prolonged and thus too severe to be relieved (e.g., long-term treatment with drugs), the deactivation of the AKT/TSC/mTOR pathway leads to autophagy and apoptosis [28]. In this study, CHOP RNAi blocking ER stress, 3-MA inhibiting autophagy and IGF-1 activating PI3K/AKT pathway could recover depression of cell viability caused by CERS1 overexpressed in glioma cells. These results indicated the causality between ER stress, autophagy, PI3K/AKT pathway and cell viability reduction in CERS1 overexpressed glioma cells. It was reported that CerS1 and C18-ceramide induced LC3B-phosphotidylethanolamine lipidation, forming LC3B-II, targeting autophagolysosomes to mitochondria and leading to lethal mitophagy and tumor suppression. CerS1 and C18-ceramide might induce activating ER stress via perturbation of cellular Ca^2+^ and ER/Golgi membrane network. In our study, ER stress might negatively regulate the PI3K/AKT pathway to induce lethal autophagy, which further increased the cell death. Sentelle et al. showed that CerS1 and C18-ceramide selectively induce non-apoptotic lethal autophagy independent of Bax, Bak, or caspase activity in HNSCC cells and tumors [16]. In our study, the lethal autophagy induced by overexpression of CERS1 was also independent of Bax (Supplementary Figure 9A, 9B) and Bcl-2 (Supplementary Figure 9C, 9D) in glioma cells.

The 5-year survival rate of glioblastoma multiforme, the most malignant glioma, is only 9.8% at best, due to difficulties in complete resection and the low sensitivity to radio/chemotherapeutic agents [45]. Therefore, it is very important to find ways to sensitize glioma cells to chemotherapy [46]. VM-26 has gradually become an effective chemotherapeutic drug for glioma due to its small molecular weight, high lipid solubility, and low cytotoxicity, all of which facilitate its passage through the blood-brain barrier [47]. Although the initial response of VM-26 is remarkable, tumor resistance develops rapidly after prolonged administration [48]. In our study, overexpression of CERS1 or adding exogenous of C18-ceramide promoted sensitivity of glioma cells to VM-26. A targeting drug delivery system might be a way to increase CERS1 specifically in tumor cells or apply C18-ceramide to tumor cells *in vivo*.

As mentioned previously, our study illustrated that C18-ceramide was lower in glioma tumor tissues compared with controls. Overexpression of CERS1 or adding exogenous of C18-ceramide inhibited cell viability and promoted cell death in glioma cells, accompanied by the activation of ER stress, induction of lethal autophagy, and inhibition of the PI3K/AKT signaling pathway. Furthermore, CERS1 or C18-ceramide combined with VM-26 had better efficacy in cell viability inhibition and cell death induction of human glioma cells than did VM-26 alone. Thus, the combined therapy of C18-ceramide may be a novel therapeutic strategy for the treatment of human glioma.

## MATERIALS AND METHODS

### Ethics statement

The samples of patients used in this study were part of the samples taken for clinical operations. In our study, informed written consent was obtained from all patients. This study was approved by the Ethics Committee of Soochow University following Declaration of Helsinki conditions.

### Materials

Antibody against β-actin was purchased from HuaAn biotechnology (Zhejiang, China). Antibodies against p-eIF2α, eIF2α, p-PERK, and PERK were purchased from Santa Cruz Biotechnology (Dallas, TX, USA). Antibodies against LC3B were purchased from Sigma-Aldrich (St. Louis, MO, USA). Antibodies against PI3K, p-AKT, and AKT were purchased from Cell Signaling Technology (Danvers, MA, USA). Antibodies against ATF-4, XBP-1, CHOP, and p62 were purchased from Abcam (Danvers, MA, USA). Anti-rabbit and anti-mouse IgG antibodies conjugated with infrared dyes were provided by LI-COR Biosciences (Lincoln, NE, USA). C18-ceramide was obtained from Avanti Polar Lipids (Alabaster, AL, USA). 3-MA and IGF-1 were purchased from Sigma-Aldrich. Cell counting kit-8 (CCK8) and BCA protein assay kit were purchased from Beyotime Institute of Biotechnology (Jiangsu, China). 7-AAD/RNase Staining Buffer, FITC Annexin V Apoptosis Detection Kit, and APC BrdU Flow Kit were acquired from BD Pharmingen (San Diego, CA, USA). The Trizol reagent was obtained from Invitrogen (Carlsbad, CA, USA). All of the RNA PCR reagents were ordered from TaKaRa (Dalian, China). SYBR Green PCR Master Mix was purchased from Applied Biosystems (Carlsbad, CA, USA). Dulbecco’s modified Eagle’s medium, antibiotics, L-glutamine, nonessential amino acids, and trypsin were purchased from Gibco (Invitrogen, UK). Fetal bovine serum was purchased from Hyclone (The Netherlands). 3-[4,5-dimethylthiazol-2-yl]2,5-diphenyl tetrazolium bromide, diethylaminoethyl Sephadex A-25, methanol, dichloromethane, isopropanol, n-hexane, iodomethane and dimethyl sulfoxide were purchased from Sigma. The C18-ceramide was dissolved with DMSO at stock concentration of 50 mM, and diluted to work concentration (20 μM) with DMEM medium. The C18-ceramide treatment was adding the C18-creamide solution (diluted with DMEM medium) to the cells, and the control was the same volume of DMSO (diluted with DMEM medium).

### Cell culture and transfection

Two human glioma cell lines, A172 and U251, were obtained from the Laboratory of Cellular and Molecular Tumor Immunology, Soochow University. They were cultured in Dulbecco’s modified Eagle’s medium, with 10% fetal bovine serum, 100 units/mL penicillin and streptomycin, 2 mM L-glutamine, and nonessential amino acids, and cells were incubated in a 37°C incubator in an atmosphere of 5% CO_2_. The cells were transfected with control vector pcDNA3.1(+) (Invitrogen), pcDNA3.1(+)/CERS1 or pcDNA3.1(+)/GFP-LC3, or negative control siRNA or CHOP RNAi siRNA using Lipofectin (Life Technologies, Grand Island, NY), according to the supplier’s instructions.

### Isolation of ceramide from human brain tissues

Total ceramides were extracted from previously frozen brain tissues (from First Affiliated Hospital of Soochow University, Suzhou, China) and glioma cell lines (A172 and U251 cell line) by extensive sonication with mixed polarity solvents. The first extraction used chloroform–methanol 1:1, v/v (solvent A), followed by extraction with isopropanol–hexane–water 65:25:20, v/v/v (solvent B) and it was replicated 4 times; the supernatant were removed before use of solvent B. Each sonication was followed by centrifugation (300×g, 5 min) to remove supernatant; all the supernatants were dried under nitrogen stream at 40°C. Ceramides were separated by anion exchange chromatography on diethylaminoethyl Sephadex A-25 as previously described [49], except that after elution of the neutral fraction with five column volumes of chloroform–methanol–water 30:60:8 (v/v/v [solvent C]), the total acid fraction were eluted by a single step of 0.8 M sodium acetate in methanol.

### Methylation of the ceramides

The neutral fractions were transferred into a conical glass vial, and dimethyl sulfoxide (150 μL) was added to each sample. Then finely powdered sodium hydroxide (ground in an agate mortar) was added to the sample solution and stirred at room temperature quickly until completely dissolved. Iodomethane (150 μL) was added with an injector, then the mixture was vortexed at room temperature for 1 h. The reaction was quenched after 2 mL deionized water was added. The permethylated products were extracted with dichloromethane (10×2 mL), and the combined dichloromethane was frozen on dry ice. The liquid phase dichloromethane was transferred into a new glass tube and dried in a centrifugal dryer.

### ESI-LIT-MS analysis of the ceramides

Electrospray ionization mass spectrometry (MS^1^ and MS^n^) was carried out in positive or negative ion mode on a linear ion trap mass spectrometer (LTQ), with a flow rate of 2 μL/min, isolation width m/z 1.5, spray voltage 3.5 kv, and at capillary temperature 300°C with injection time 50.00 ms. All ions were detected as sodium adducts. Each ceramide sample was dissolved in methanol (100-500 μL), then the general MS^1^ was measured in ESI-LIT-MS, and the MS^n^ spectra was obtained from multistep fragmentation via the MS^n^ pathway as previously described [50, 51]. The ceramide and sphingolipid quantification was performed as described previously and normalized to total protein levels [15, 52].

### Cell viability assay

The CCK-8 assay was used to measure the effect of ceramide on cell viability. U251 and A172 cells were seeded at 5000 per well in 96-well plates. The supernatant fluid was removed, and CCK-8 was added to each well filled with 110 μL medium (10 μL CCK-8, Beyotime Institute of Biotechnology). After incubation for 2 h, the absorbance was measured at 450 nm with a microplate reader (Synergy HT, Bio-tech, USA).

### Apoptosis detection by annexin V-FITC/7-AAD staining

Cells were seeded in culture dishes and allowed to adhere overnight. The cells were harvested, washed, and resuspended in 1× binding buffer. In the next step, the cells were stained at a ratio of per 100,000 cells; staining of cells with 5 μL of FITC-Annexin V (556420, BD Pharmingen) and 5 μL of 7-AAD (50 μg/mL) was done for 15 min. Then, 200 μL of 1× binding buffer was added to each sample. Unstained, untreated cells were also included as controls. A minimum number of 10,000 events was acquired for each replicate using flow cytometry. A quadrant statistic was used to measure the population of viable, early apoptotic, late apoptotic, and secondary necrotic cells. These different populations were determined by estimating quantitatively the cells that were stained with AnnexinV/7-AAD.

### Gene expression microarray analysis

Total RNA was extracted using Trizol reagent according to the manufacturer’s instructions and stored at –80°C until quality tests and microarray analyses. Gene expression microarray experiments were performed using the SurePrint G3 Human Gene Expression 8×60 K (Agilent Technologies) by CapitalBio Corporation (Beijing, China) according to the standard protocols.

### qRT-PCR analysis

Total RNA was extracted with Trizol reagent, and RNA concentration was measured by a microplate reader. For all samples, the A260/A280 ratio was between 1.8 and 2.0. The synthesis of first-strand cDNA was performed by using TaKaRa RNA reverse transcription reagents. The primers were designed by Primer-Blast (www.ncbi.nlm.nih.gov/tools/primer-blast/). Then PCR reactions with three replicates for each cDNA sample were carried out using 2×SYBR Green PCR master Mix on the real-time PCR system (Applied Biosystems). The thermal cycle profile was as followings: 95°C for 5 min, followed by 95°C for 15 s, 60°C for 60 s, and 72°C for 15 s for 40 cycles. Then the melting curves were generated to confirm the presence of a single amplicon. The 2^∆∆CT^ was calculated to assess the relative mRNA expression.

### Western blot assay

Cells treated with reagents for indicated times were lysed in RIPA buffer supplemented with a proteinase inhibitor (10 mg/mL aprotinin, 10 mg/mL phenyl-methylsulfonyl chloride, and 50 mM sodium orthovanadate). Cell extracts were quantified using the BCA protein assay kit, and equal amounts of proteins were separated by SDS-PAGE. Gels were then transferred to PVDF membranes, blocked with 5% non-fat dry milk, and incubated with the primary antibody solution. Alternatively, primary antibodies were diluted in 5% BSA in PBST overnight at 4°C. The membrane was washed with PBST and incubated with secondary antibody conjugated with infrared dyes for 2 h at room temperature. Finally, the protein bands were detected by Odyssey infrared image system (LI-COR, Lincoln, NE, USA), and the protein expression levels were quantified by Image Studio software (Lincoln, NE, USA).

### Statistical analysis

All the results were expressed as the mean ± standard deviation (SD) of data obtained from three separate experiments and determined using t-test to compare with controls. The n numbers show independent experiments. P < 0.05 was considered as statistically significant (*P < 0.05, **P < 0.01, ***P < 0.001). All statistical analyses were evaluated using SPSS 13.0 software (New York, NY, USA).

## SUPPLEMENTARY MATERIALS FIGURES



## Abbreviations

ATF-6: activating transcription factor 6
BA1: bafilomycin A1
CERS1: ceramide synthase 1
CHOP: C/EBP homologous protein
CNS: central nervous system
C18-ceramide: ceramide with an 18-carbon–containing fatty acid chain
eIF2α: eukaryotic translation initiation factor 2α
ER: endoplasmic reticulum
ESI-LIT-MS: electrospray ionization linear ion trap mass spectrometry
HNSCC: head and neck squamous cell carcinoma
IGF-1: insulin-like growth factor 1
IRE1: inositol-requiring kinase 1
PERK: PKR-like ER kinase
VM-26: teniposide
XBP-1: X-box binding protein 1
