# Supplementary material for: Overexpression of ceramide synthase 1 increases C18-ceramide and leads to lethal autophagy in human glioma
Source: Oncotarget. 2017 Oct 23;8(61):104022–36. doi: 10.18632/oncotarget.21955 (PMC5732784; doi:10.18632/oncotarget.21955)
Supplement: Supplementary file 1 [file oncotarget-08-104022-s001.pdf]

# Overexpression of ceramide synthase 1 increases C18-ceramide and leads to lethal autophagy in human glioma

## SUPPLEMENTARY MATERIALS

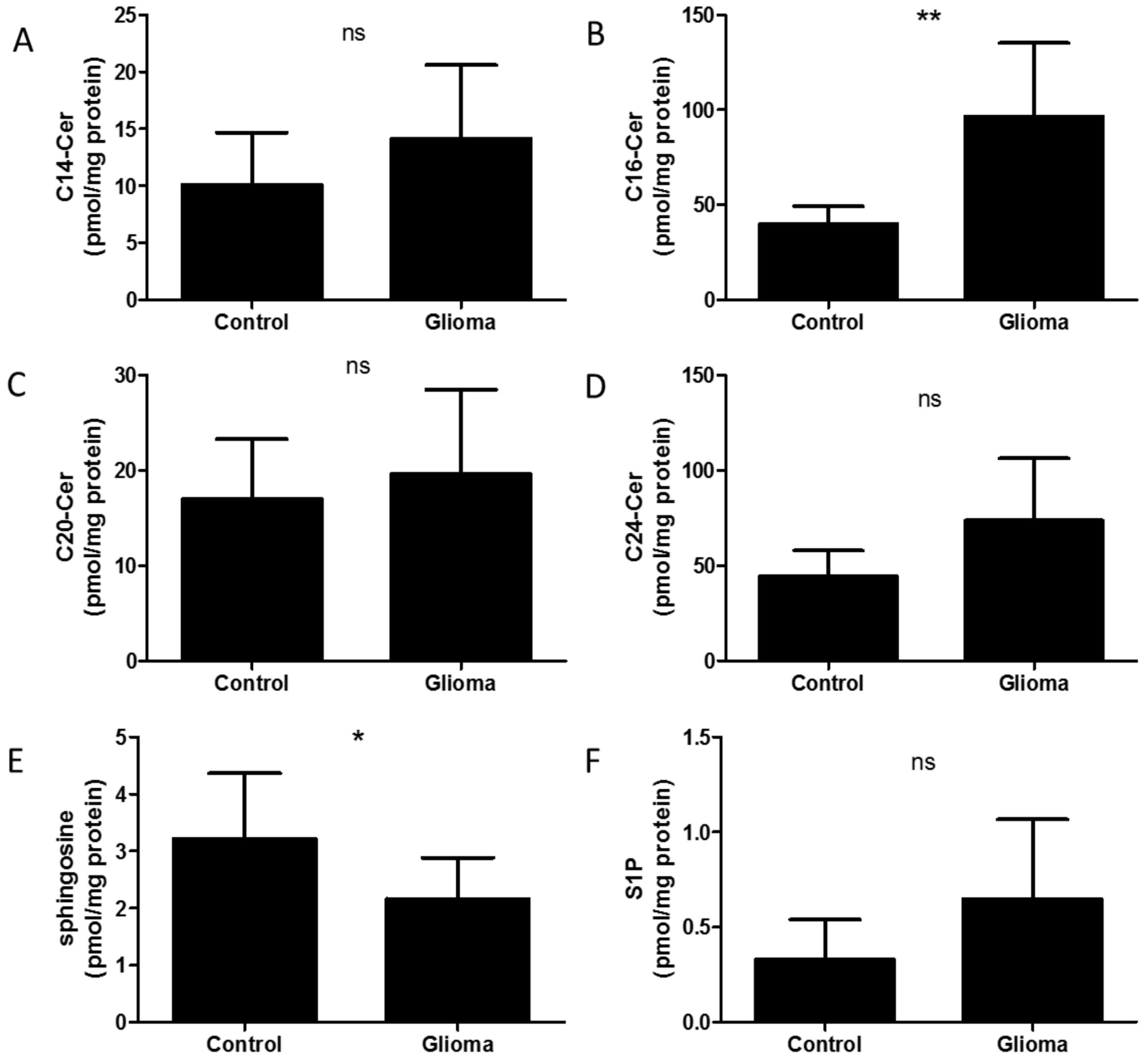


**Supplementary Figure 1:** Relative quantification of C14-ceramide **(A)**, C16-ceramide **(B)**, C20-ceramide **(C)**, C24-ceramide **(D)**, sphingosine **(E)** and S1P **(F)** in the tissue samples of controls and glioma. Data represent the tissue samples from controls (n = 5) and glioma (n = 14). Statistical significance between glioma and controls was analyzed using the two-tailed Student's t-test of means. Values represent the means ± SD, n = 3 independent experiments. Compared with control, $^{*}P < 0.05$, $^{**}P < 0.01$, *ns*, no significant difference.

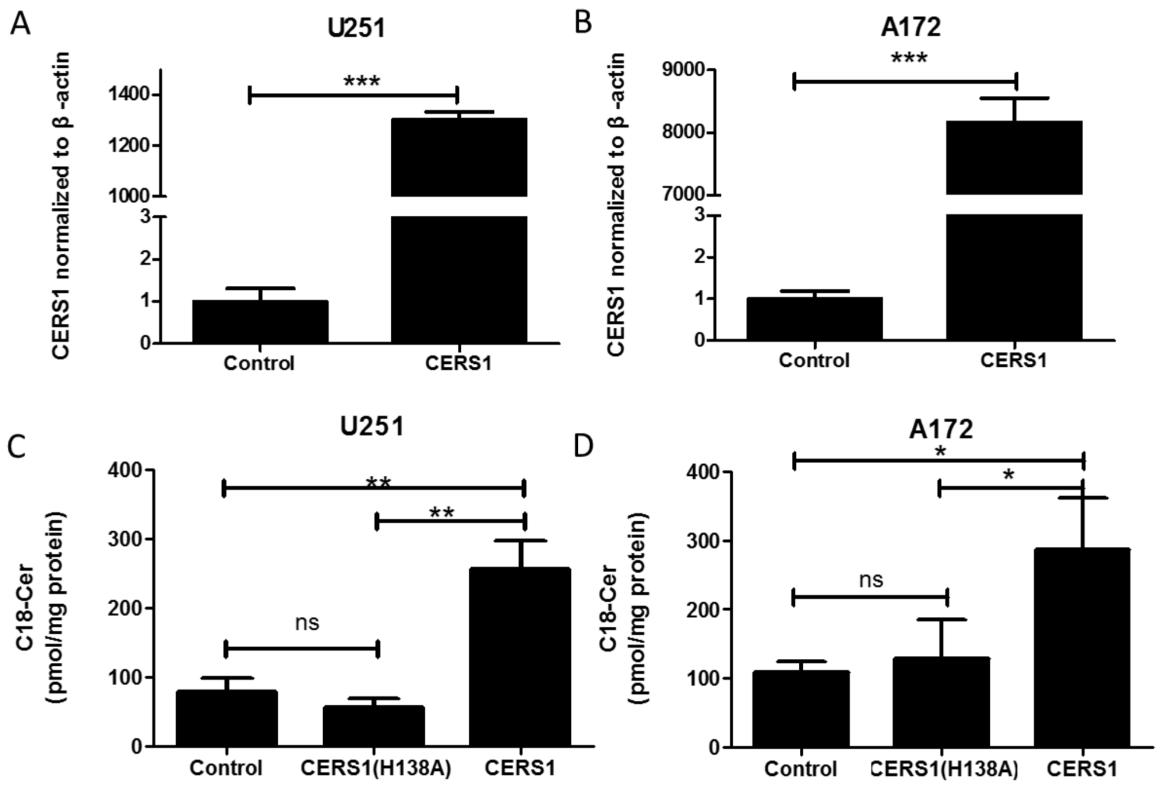


**Supplementary Figure 2: (A)** The qRT-PCR results of CERS1 mRNA levels in CERS1 overexpression U251 cells compared with controls. **(B)** The qRT-PCR results of CERS1 mRNA levels in CERS1 overexpression A172 cells compared with controls. **(C)** Relative quantification of C18-ceramide (m/z 630) in catalytically inactive CERS1 (H138A) and CERS1 overexpression U251 cells compared with controls. **(D)** Relative quantification of C18-ceramide (m/z 630) in catalytically inactive CERS1 (H138A) and CERS1 overexpression A172 cells compared with controls. Statistical significance between CERS1 and controls was analyzed using the two-tailed Student's t-test of means. Values represent the means ± SD, n = 3 independent experiments. Compared with control, $^{*}P < 0.05$, $^{**}P < 0.01$, $^{***}P < 0.001$.

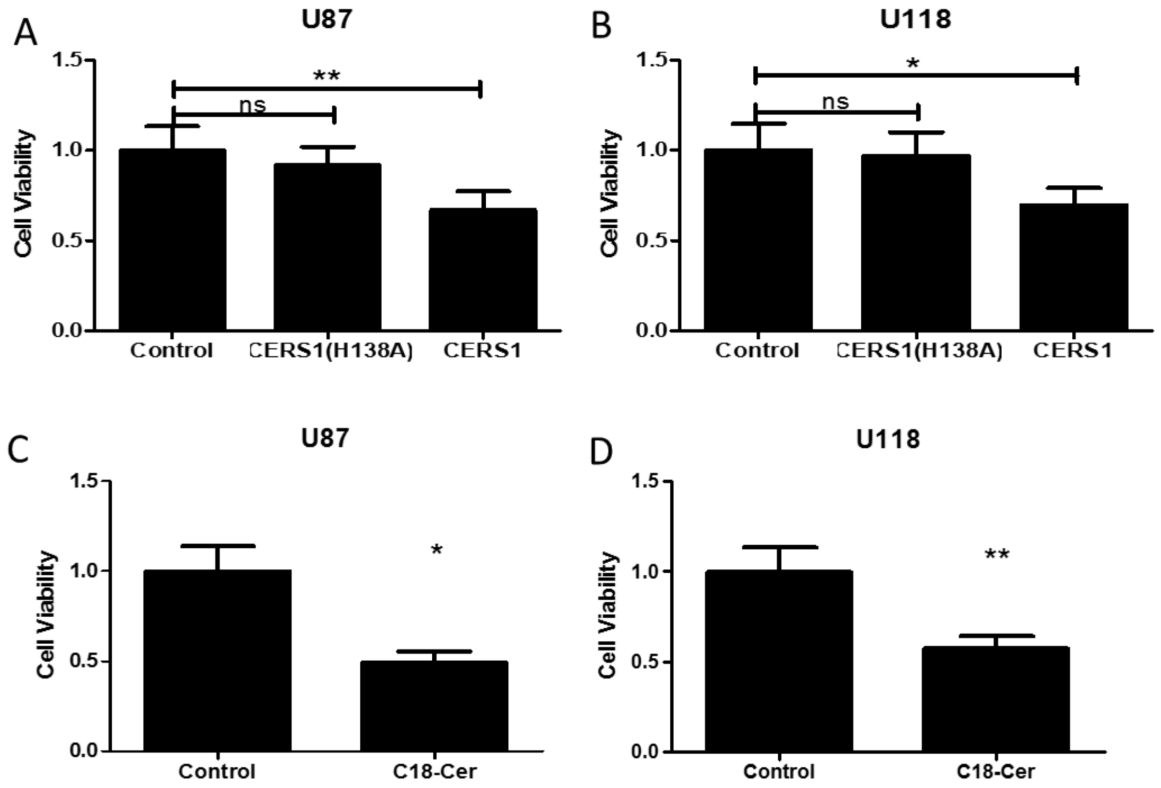


**Supplementary Figure 3:** Effect of catalytically inactive CERS1 (H138A) and CERS1 overexpression on the cell viability of U87 **(A)** and U118 **(B)** cells for 48h. Effect of exogenous C18-ceramide (20 µM) on the cell viability of U87 **(C)** and U118 **(D)** cells for 48h. Statistical significance between CERS1/C18-Cer and controls was analyzed using the two-tailed Student's t-test of means. Values represent the means ± SD, n = 3 independent experiments. Compared with control, $^{*}P < 0.05$, $^{**}P < 0.01$, *ns*, no significant difference.

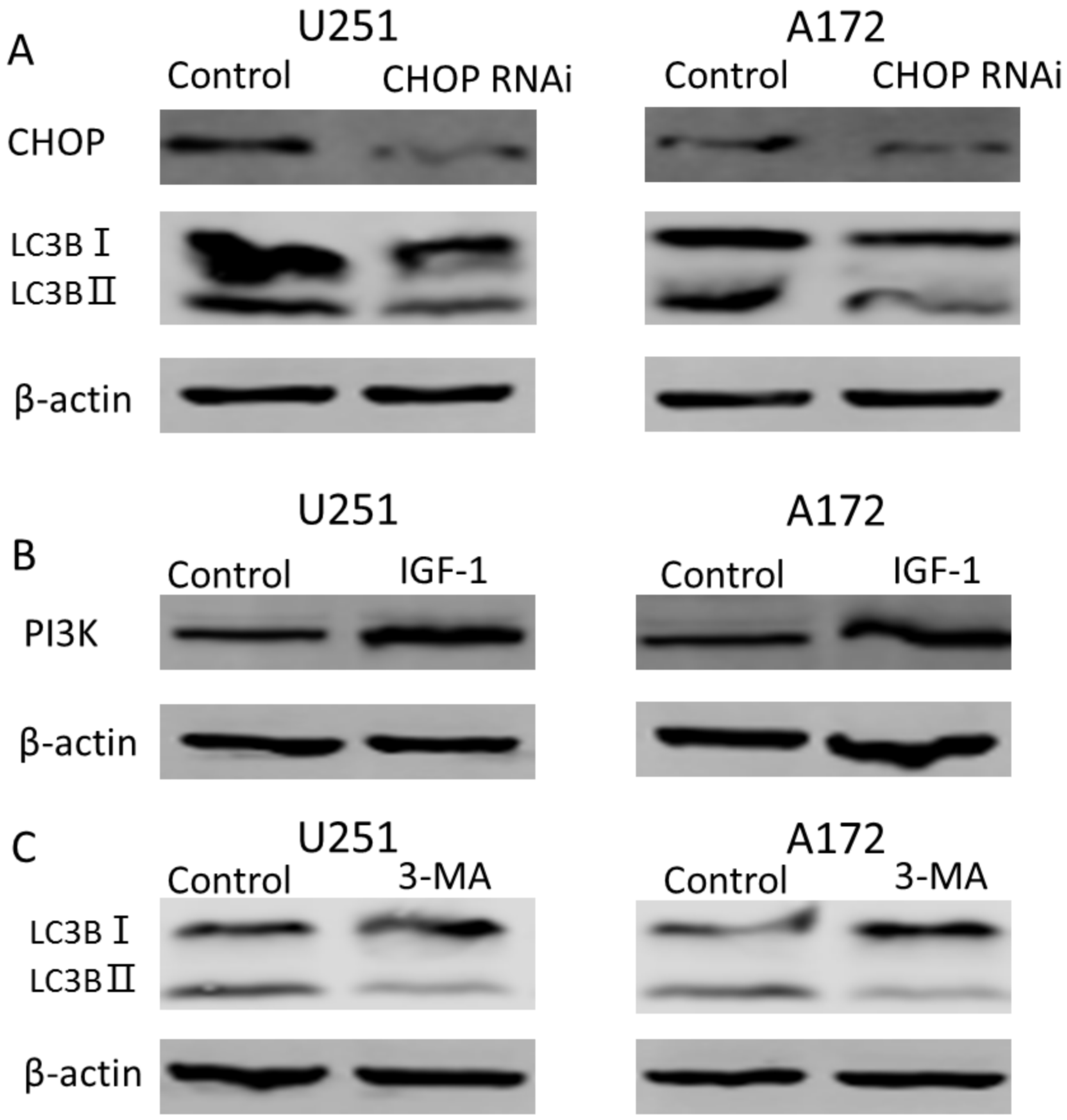


**Supplementary Figure 4:** Control studies to validate CHOP knockdown in response to siRNA and effects of CHOP knockdown on autophagy induction (LC3) were measured **(A)**, PI3K activation in response to IGF1 **(B)**, and inhibition of autophagy (LC3) in response to 3-MA **(C)** were performed by western blot.

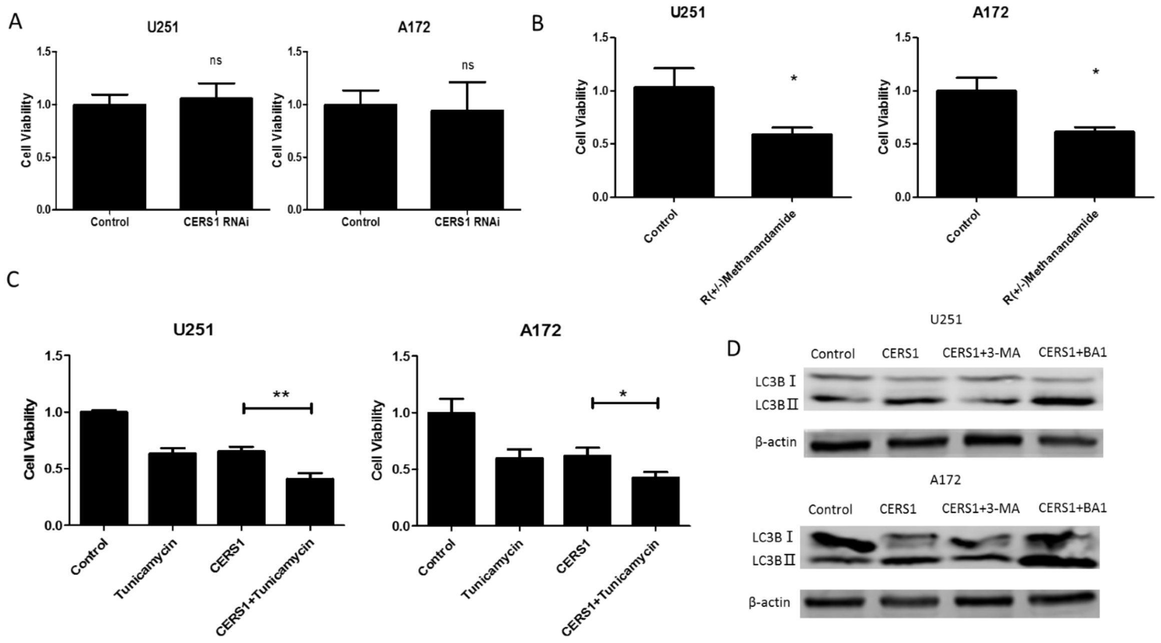


**Supplementary Figure 5: (A)** Effect of CERS1 RNAi on the cell viability of U251 and A172 cells. **(B)** Effect of R (+/-) Methanandamide (10 μM) on the cell viability of U251 and A172 cells for 48h. **(C)** Effect of Tunicamycin (1 μg/ml) on the cell viability of U251 and A172 cells for 48h. **(D)** Effect of Bafilomycin A1 (BA1) (100 nM) on the autophagy (LC3) of U251 and A172 cells for 48h. Statistical significance between glioma and controls was analyzed using the two-tailed Student's t-test of means. Values represent the means ± SD, n = 3 independent experiments. $^{*}$ $P < 0.05$, $^{**}$ $P < 0.01$, *ns*, no significant difference.

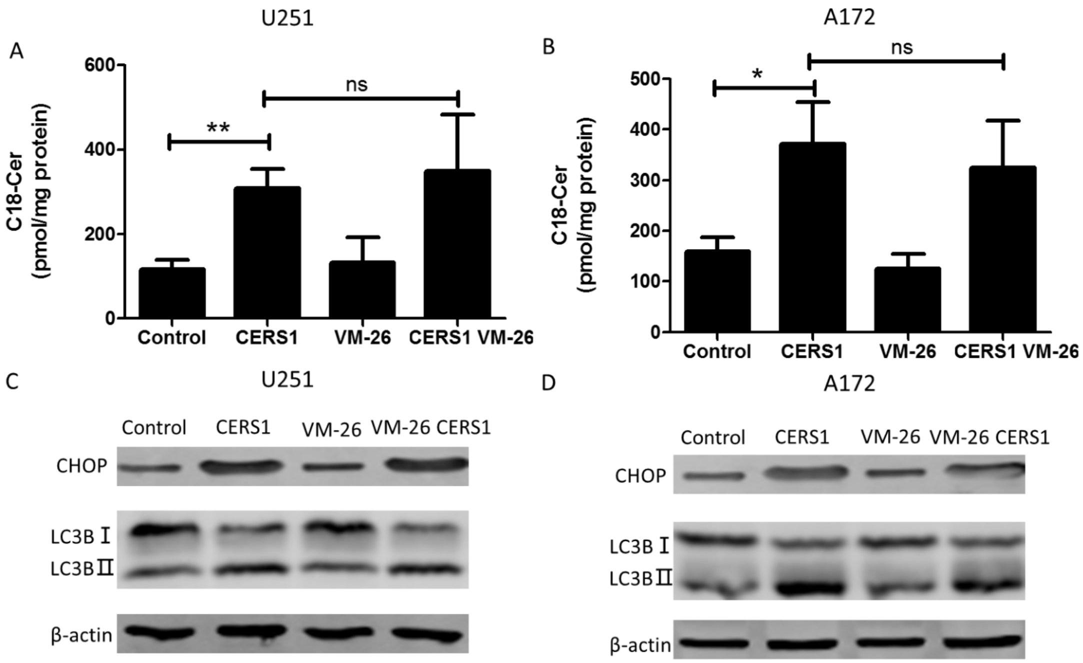


**Supplementary Figure 6:** The effects of CERS1 overexpression and VM-26 combination treatment on C18-ceramide levels in U251 cells **(A)** and A172 cells **(B)**, as well as on induction of ER stress (CHOP) and autophagy (LC3) in U251 cells **(C)** and A172 cells **(D)** were measured. Statistical significance between glioma and controls was analyzed using the two-tailed Student's t-test of means. Values represent the means ± SD, n = 3 independent experiments. $^{*}$ $P < 0.05$, $^{**}$ $P < 0.01$, *ns*, no significant difference.

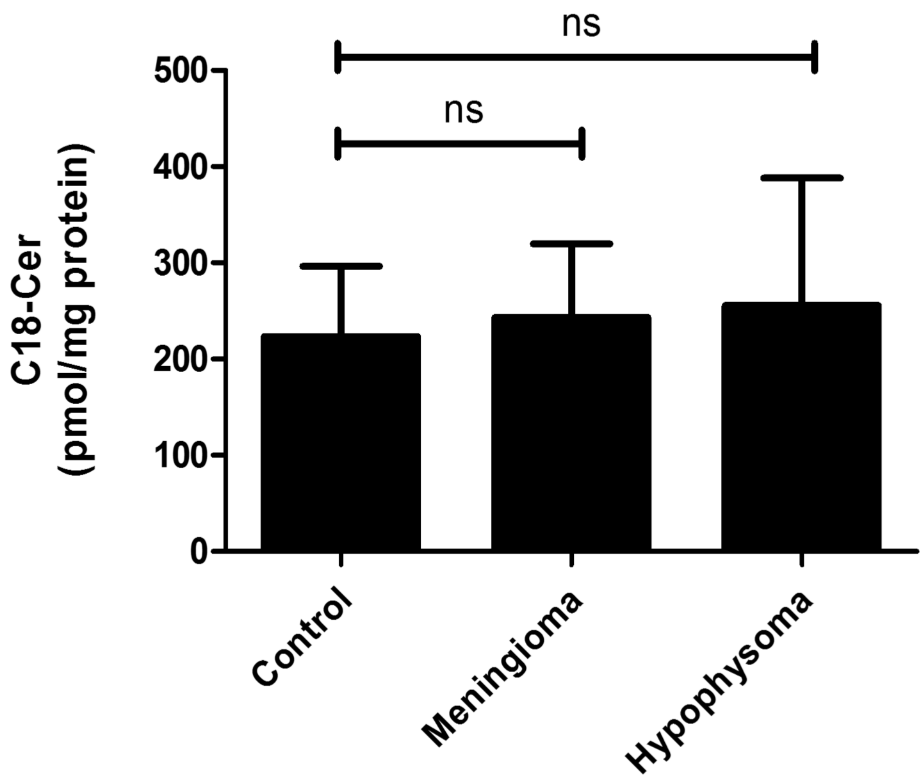


**Supplementary Figure 7: Relative quantification of C18-ceramide in the tissue samples of controls, meningioma patients and hypophysoma patients.** Data represent the tissue samples from control (n = 5), meningioma (n = 6) and hypophysoma (n = 16). Statistical significance between meningioma patients, hypophysoma patients and controls was analyzed using the two-tailed Student's t-test of means. Values represent the means ± SD. Compared with control, *ns*, no significant difference.

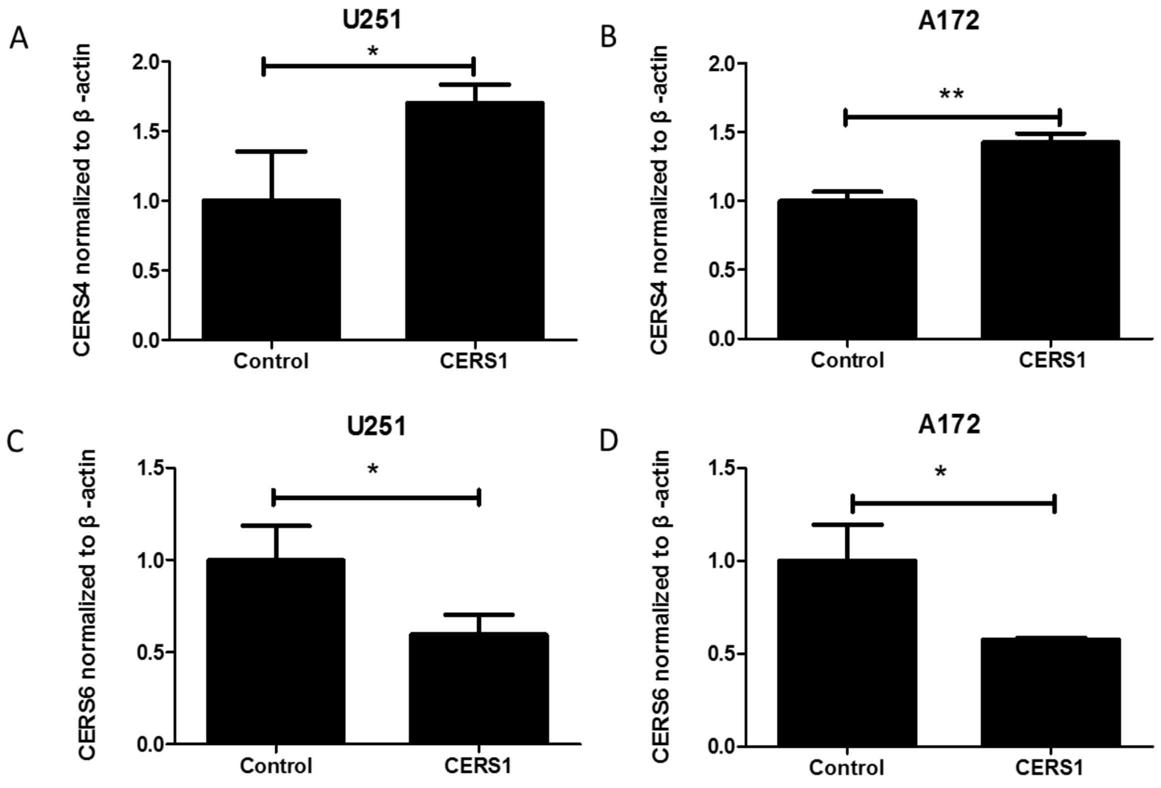


**Supplementary Figure 8: (A)** qRT-PCR results of CERS4 mRNA levels in CERS1 overexpression U251 cells compared with controls. **(B)** qRT-PCR results of CERS4 mRNA levels in CERS1 overexpression A172 cells compared with controls. **(C)** qRT-PCR results of CERS6 mRNA levels in CERS1 overexpression U251 cells compared with controls. **(D)** qRT-PCR results of CERS6 mRNA levels in CERS1 overexpression A172 cells compared with controls. Statistical significance between CERS1 and controls was analyzed using the two-tailed Student's t-test of means. Values represent the means ± SD, n = 3 independent experiments. Compared with control, $^{*}P < 0.05$, $^{**}P < 0.01$.

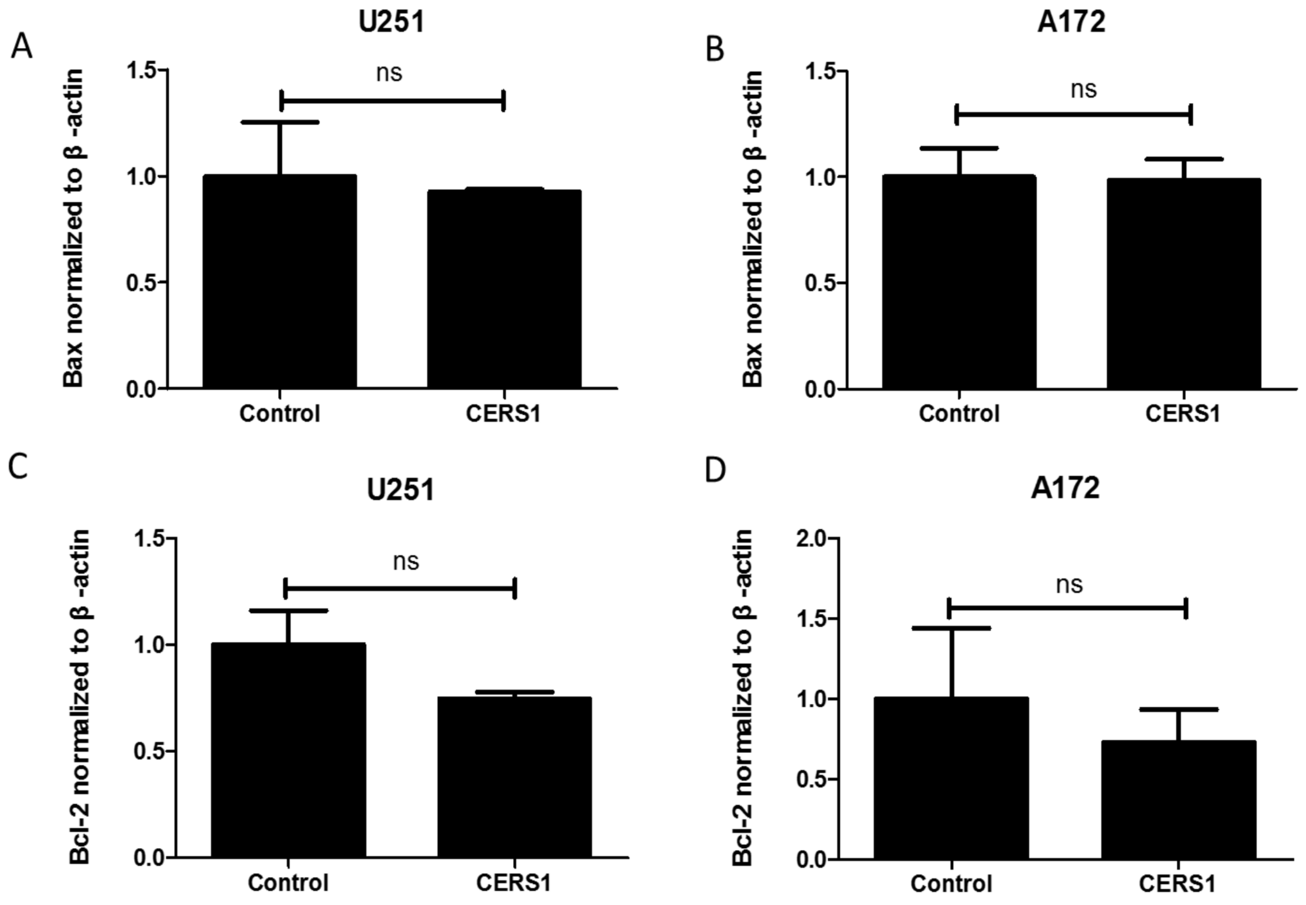


**Supplementary Figure 9:** **(A)** qRT-PCR results of Bax mRNA levels in CERS1 overexpression U251 cells compared with controls. **(B)** qRT-PCR results of Bax mRNA levels in CERS1 overexpression A172 cells compared with controls. **(C)** The qRT-PCR results of Bcl-2 mRNA levels in CERS1 overexpression U251 cells compared with controls. **(D)** The qRT-PCR results of Bcl-2 mRNA levels in CERS1 overexpression A172 cells compared with controls. Statistical significance of differences between CERS1 and controls was analyzed using the two-tailed Student's t-test of means. Values represent the means ± SD, n = 3 independent experiments. Compared with control; *ns*, no significant difference.
